# Examining grounded theory through the lens of rationalist epistemology

**DOI:** 10.1007/s10459-018-9849-7

**Published:** 2018-08-09

**Authors:** Mathieu Albert, Maria Mylopoulos, Suzanne Laberge

**Affiliations:** 1 Wilson Centre and Department of Psychiatry, University of Toronto, Toronto, ON Canada; 2 Wilson Centre, MD Program and Department of Paediatrics, University of Toronto, Toronto, ON Canada; 3 School of kinesiology and physical activity sciences, Université de Montréal, Montréal, QC Canada

## Abstract

The objective of scientific, or more broadly, academic knowledge is to provide an understanding of the social and natural world that lies beyond common sense and everyday thinking. Academics use an array of techniques, methods and conceptual apparatuses to achieve this goal. The question we explore in this essay is the following: Does the grounded theory approach, in the constructivist version developed by Kathy Charmaz, provide the necessary methodological tools for the creation of knowledge and theories beyond everyday thinking? To conduct our analysis, we have drawn on the rationalist epistemology originally developed by Gaston Bachelard and taken up a few decades later by Pierre Bourdieu and colleagues to look at the epistemological foundation of the CGT methods as defined by Charmaz. We focussed on two distinctive epistemological features characterising constructivist grounded theory (CGT): the use of inductive reasoning to generate interpretative theory; and the primacy of subjectivity over objectivity as the preferred path to knowledge making. While the usefulness of CGT for conducting qualitative research and understanding the perspective of social actors has been acknowledged by scholars in health professions education research and other research areas, the inductivist logic on which it draws raises questions concerning the nature of the knowledge yielded by this approach. As we argue in this article, it is still unclear in what way the interpretative theory generated by CGT is not a duplication of everyday thinking expressed through meta-narratives. It is also unclear how the understanding of social phenomena can be refined if the use of inductive procedures logically implies the creation of a new theory each time a study is conducted. We engage with these questions to broaden the epistemological conversation within the health professions education research community. It is our hope that scholars in the field will engage in this epistemological conversation and advance it in new directions.

## Introduction

It is uncontroversial to affirm that the objective of scientific, or more broadly, academic knowledge is to provide an understanding of the social and natural world that lies beyond common sense and everyday thinking. Academics use an array of techniques, methods and conceptual apparatuses to achieve this goal. The question we wish to explore in this essay is the following: Does the grounded theory approach, in the constructivist version developed by Charmaz (1990, 2005, 2008a, b, 2014a, b, 2017), provide the necessary methodological tools for the creation of knowledge and theories beyond everyday thinking? To conduct our analysis, we will focus on two distinctive epistemological features characterising constructivist grounded theory: the use of inductive reasoning to generate interpretative theory; and the primacy of subjectivity over objectivity as the preferred path to knowledge making.

Our interest in grounded theory has been prompted by its growing popularity in several research areas (Tavory and Timmermans 2014), including health professions education research. Grounded theory, and its revised version, constructivist grounded theory (CGT), is now accepted as a legitimate research practice: over 50 articles were published between 2013 and 2017 in the journals *Medical Education* and *Advances in Health Sciences Education* using this approach, and several key essays have cogently delineated its epistemological foundation, analytical procedures and historical roots (Harris 2003; Kennedy and Lingard 2006; Watling and Lingard 2012).

This reflection article adds to the debate initiated by sociologists and education scholars two decades ago concerning the nature of the knowledge yielded by grounded theory (Buravoy 1998; Kelle 2007; Miller and Fredericks 1999; Tavory and Timmermans 2014; Thomas and James 2006). Building on insights from the philosophy of science, psychology and sociology, these scholars challenged one of the central assumptions of grounded theory: that a theory can be inductively derived from empirical data. While “induction has an important place in research,” note Tavory and Timmermans, “its strength is not in generating new theories” (2014, p. 15). Likewise, Buravoy stresses that “theories do not spring tabula rasa from the data but are carried forward through intellectual debate and division” (1998, p. 16). Kelle echoes these criticisms, contending that “inductivist rhetoric” should be abandoned (2007, p. 154) in order to make space for a broader mix of reasoning in theory generation. As much as grounded theory may have become a quality benchmark for many qualitative researchers (Tavory and Timmermans 2014), the critical stance taken by several scholars suggests that its capacity to deliver on its promise (generate theories) is still considered speculative.

In this article, we examine the constructivist version of grounded theory developed by Charmaz through the lens of rationalist epistemology. While CGT and rationalism originate from very different epistemologies, both aim at delineating the proper route to generating theoretical knowledge. For this reason, the similarities in their fundamental goal, it is legitimate to examine the nature of the knowledge generated by the CGT methodology through the lens of a rationalist perspective, just as it would be legitimate to do the reverse. Querying an epistemological position (constructivism) from a different epistemological position (rationalism) can lead to fruitful debates and bring attention to ideas that may otherwise be taken for granted when situated within one epistemology. Working through questions and critiques can enhance researchers’ own understandings of their epistemological position and rationale for choosing certain theoretical frameworks over another.

In what follows, we will outline how a rationalist epistemology distinguishes scientific/academic knowledge from commonsensical knowledge and everyday thinking. We will then consider grounded theory from this epistemological angle, drawing on the epistemological position articulated by the French sociologists Pierre Bourdieu, Jean-Claude Chamboredon and Jean-Claude Passeron in their seminal book *The Craft of Sociology: Epistemological Preliminaries* (1991 [1968]). In this work, Bourdieu and colleagues build on Gaston Bachelard’s philosophy of science, referred to as rationalism (Bachelard 1985 [1934]). They delineate the conditions under which a claim about the social and the natural world can be said to have (or at least, can have aimed to) overcome commonsensical thinking. As Bourdieu and colleagues explain, achieving this break implies that researchers engage in the construction of “facts.” It is through these constructed facts, they argue, that we can gain a scientific knowledge of the social and natural world (Bourdieu et al. 1991, p. 57). In the next section, we explain what rationalists mean by “constructing facts” and outline the steps involved in this process.

## The construction of social facts: the rationalist position

Bourdieu et al. (1991) conceptualised the construction of social/scientific facts as a three-step process: (1) social/scientific facts are “won” (*conquis*) over everyday thinking (i.e., taken-for-granted assumptions, ideology, preconstructed objects, commonsensical explanations); (2) they are “constructed” (*construit*) by researchers through theories, concepts and methodological apparatuses; and (3) they are “confirmed” (*constaté*) through empirical verification (Bourdieu et al. 1991, p. 57). These steps do not represent three distinct operations, but a single procedure comprising three interconnected components. For example, theorisation entails an act of abstraction (i.e., the conceptual construction of the research object) and a break from everyday thinking (theorisation can only occur when one breaks from commonsensical explanations).

This three-step procedure represents what both Bachelard and Bourdieu et al. (1991) call the “epistemological break”: creating a rupture with ready-made perceptual categories allowing the unlocking of a research/theoretical space conducive to scientific investigation. Put more simply, what rationalists claim is that theorisation comes first (based on extant empirical and theoretical knowledge), data collection, second. Theories and concepts serve as the anchor and the driving force for the research process, from the construction of the research object to the construction of the social/scientific fact. They serve to break away from everyday thinking, and to make visible—both empirically and conceptually—the underlying structures, processes, and mechanisms shaping the social (and the natural) world. Examples of structures, processes, and mechanisms commonly studied in the social sciences include power struggles, boundary-work, social class reproduction, stigmatisation, symbolic violence and hierarchisation processes.

Bourdieu et al. explain what an epistemological break entails. “One needs to have broken free from the phenomenal resemblances [i.e., from what reality seems to be when perceived through commonsensical cognitive categories] in order to construct the deep analogies [i.e., the underlying structures and processes similarly active in multiple settings], and this break with apparent relations [i.e., with false evidence] presupposes the construction of new relations among the appearances [i.e., among the structures, mechanisms and processes brought to light through theory and concept]” (Bourdieu et al. 1991, p. 57).

The centrality of the act of theory-construction in the science-making process puts rationalism in sharp contrast with empiricism (Bourdieu et al. 1991, p. 38). Indeed, in the rationalist perspective, a fact is not a tangible observation, neither something pre-existing waiting to be discovered, nor an event that one can see and date (such as the 1789 French Revolution or the first landing on the moon in July 1969). A fact is a conceptual construction at which academics arrive through a series of cognitive and methodological procedures, which the notion of epistemological break encapsulates.

Performing an epistemological break is all the more critical for social scientists, as they are embedded in the social world they are investigating (i.e., they are part of their research object). The sense of familiarity social scientists have with the social world makes them vulnerable to inadvertently introducing preconstructed objects through everyday language and cognitive categories (which carry embedded signification) into their own conceptualisation. Their situation, in this regard, contrasts with that of natural scientists, who stand outside their research object; their theories and hypotheses cannot be shaped by language and cognitive categories entrenched within natural objects as these objects do not talk and think. These objects (such as plants, molecules, or stars) do not inherently carry with them a socially constructed symbolic system (such as the notions of curriculum, professionalism and evidence do) and therefore cannot surreptitiously impose a pre-determined signification upon the researchers. Social scientists are faced with their own double epistemological obstacle (within themselves as social actors and within their research object as socially constructed), which they need to overcome by performing an epistemological break.

“Sociologist[s’] familiarity with [their] social universe is the epistemological obstacle *par excellence*,” write Bourdieu and colleagues, “because it continuously produces fictitious conceptions or systematizations and, at the same time, the conditions of their credibility. The sociologist’s struggle with spontaneous sociology is never finally won, and he must conduct unending polemics against the blinding self-evidences which all too easily provide the illusion of immediate knowledge (…)” (Bourdieu et al. 1991, p. 13).^1^

This is not to say, of course, that natural scientists are immune to the influence of economic, political and cultural forces. As social actors, they are embedded in the social world, and as such make decisions that reflect this embeddedness (see Bourdieu 2004; Albert and McGuire 2014).

The task of theoretically constructing the research object is not based on the rejection of the existence of an external reality—a position typically upheld by post-modern scholars (see Johnson and Duberley 2000, on post-modern epistemology). The purpose of this task is to raise researchers’ awareness of the preconstructed objects they may unconsciously be carrying with them through unexamined assumptions, whether it be through everyday vocabulary, ideologies, false evidence, etc. Given that the world is only accessible through cognitive categories and never directly, rationalists state that categories unconsciously internalised throughout one’s social existence need to be made explicit and bracketed in order to give way to new categories (theory-based), consciously and reflexively constructed and sufficiently robust to ward off commonsensical thinking.

In summary, the key feature of rationalist epistemology is that the path towards knowledge creation does not go from empirical observation to theory construction, but rather the opposite, from theories and concepts to empirical examination. Theorisation is an act of creativity building on knowledge generated by previous research work (see Kelle 2007; Tavory and Timmermans 2014, on abductive reasoning; Layder 1982, on the pitfall of empiricism in grounded theory). Theory accomplishes two interconnected functions: (1) it breaks away from commonsensical thinking (i.e., preconstructed objects, ideology, taken-for-granted assumptions, false evidence) and, by doing so, (2) it opens up a space where new relationships between elements can be conceptually constructed and observed. This is how, for rationalists, theory acquires its explanatory power and why it focuses on the underlying processes, mechanisms and structures rather than on fleeting visible phenomena. This is also why rationalists, while acknowledging the idiosyncrasies of each researched phenomenon, also see commonalities between them as they may be fuelled by similar processes.

## Grounded theory through the rationalist lens

In what follows, we examine the grounded theory epistemology using a rationalist lens. Our aim is to characterise the nature of the knowledge and theories generated by this research approach and determine the extent to which they generate an understanding of social life that is different from everyday thinking. We will draw on Kathy Charmaz’s constructivist grounded theory (CGT). Charmaz has become one of the most influential figures in grounded theory research in recent years (see for example, 1990, 2005, 2008a, b, 2014a, b, 2017). Her reinterpretation of traditional grounded theory developed by Glaser and Strauss (1967) has inspired numerous qualitative researchers, including in health professions education research. As mentioned in the introduction, our analysis focuses on the use of inductive reasoning to generate interpretative theory (vs. explanatory theory), and on the primacy of subjectivity over objectivity in knowledge making. The question of the relationship between induction and deduction, and between subjectivity and objectivity traverse all epistemological theories, including rationalism, positivism, relativism, etc., and constitute a fundamental issue in any epistemological debate. Using a rationalist lens, we focus here on how CGT articulates these relationships in support of its research procedures.

It should be noted that our analysis does not address the procedural component of CGT: the tasks related to data collection and data organisation. In our view, the use of these technical procedures does not necessarily entail the adoption of the CGT specific epistemology. Indeed, many qualitative social scientists, including those with a rationalist lens, use key features of these procedures without sharing CGT’s epistemology, as the procedural components are relatively standard.

### Inductive reasoning: from data to theory

Charmaz’s recent publications (2008a, 2014a, 2017) suggest that the relationship between CGT and existing theories is ambiguous. On the one hand, following the tenets of Glaser and Strauss’s grounded theory methods (1967), Charmaz asserts the primacy of inductive procedures for theory building, according to which data collection should proceed without pre-existing theory. She writes, “grounded theory methods consist of a systematic approach to qualitative inquiry for the purpose of theory construction. This method begins with inductive data and adopts key strategies for doing research. (…) Grounded theorists value (…) developing fresh concepts and theories over applying received theory (…)” (Charmaz 2017, p. 1–2). On the other hand, she affirms that researchers cannot engage in research without building on prior theoretical knowledge, and yet they should be wary of this knowledge. Charmaz explains, “constructionists advocate recognizing prior knowledge and theoretical preconceptions and subjecting them to rigorous scrutiny” (Charmaz 2008a, p. 402). “We are not led blindly by existing research and theoretical literatures. Instead, we dissect and assess them with critical skepticism. Theoretical agnosticism involves doubt” (Charmaz 2017, p. 5).

While the first part of Charmaz’s position is clear (she favors induction), the second part appears somewhat equivocal. On the one hand, Charmaz seems to be making the argument that any individuals engaging in research cannot do so without holding prior knowledge and theory—which, in our view, is inevitable—but on the other hand, she contends that they should be treating this knowledge as a potential threat to the proper collection and analysis of the data. As Charmaz stresses, this “prior knowledge,” if not “scrutinised,” “dissected,” and “assessed” with “critical skepticism,” could be an impediment to “thinking afresh” and looking at “studied life from multiple vantage points” (Charmaz 2014a, p. 244). Our understanding of Charmaz’s position is that, ultimately, she seems ambivalent whether theory is a useful tool to unearth and comprehend hidden social mechanisms. Her inductivist epistemology seems to lead her to be doubtful about the heuristic role of theory.

Following this reasoning, two questions come to mind: (1) How can data be transformed into theory and thereby generate an understanding of the social world? (2) How can data contain their own sense-making principle?

Charmaz’s answer to these questions is twofold: (1) researchers play a central role in developing theories through the iterative process of collecting data, coding it and creating categories; and (2) the type of theory CGT generates is not explanatory, but interpretative—i.e., based on the researchers’ sense-making work. In sum, theory can be derived from data because of the close engagement of the researcher with the studied material; it is the researcher who creates the theoretical understanding of the data through an interpretative process (for an example of such a theorisation process, see Charmaz 2017, pp. 3–5, 2014a, pp. 42–72). As Charmaz affirms, “the theory *depends* on the researcher’s view; it does not and cannot stand outside of it” (Charmaz 2014a, p. 239) [emphasis in original]. She provides further details about the nature of the theory yielded by CGT, stating that this approach “emphasizes interpretation and gives *abstract understanding* greater priority than explanation” (Charma 2014a, p. 230) [emphasis in original]. She adds, “Interpretative theories aim to understand meanings and actions and how people construct them. (…) Interpretative theory calls for the imaginative understanding of the studied phenomenon” (Charmaz 2014a, p. 231).

In light of Charmaz’s description, theories developed through CGT procedures are equivalent to meta-narratives. These narratives are the result of the researcher’s personal interpretation. Indeed, when one affirms, as Charmaz does, (1) that new knowledge (new theory) is entirely dependent on the researcher’s view, (2) that one should be wary of extant theories, and (3) that induction should principally guide inquiry procedures, it is reasonable to conclude that the production of knowledge is likely to be shaped, in a fundamental way, by the researcher’s world view. The kind of knowledge produced (from the framing of the research question to the analysis of the data) becomes inseparable from the researcher’s internalised socio-cultural schemes, which necessarily carry taken-for-granted assumptions, ideology, pre-constructed objects, etc. What the researcher sees, or is able to see, is shaped by his/her particular social experiences and corresponding schemes of thought. The researcher’s gaze is unlikely to be informed by theory and prior knowledge, but rather by his/her position in the social space (social class, ethnicity, gender, etc.) and corresponding attitudes and beliefs.

According to a rationalist perspective, discarding the idea of building on previous theoretical knowledge to perform research, as Charmaz seems to suggest, cannot free one’s mind from presumably constraining preconceptions. Rather, it exposes one to unexamined beliefs and ways of thinking (i.e., taken-for-granted assumptions, etc.). Inductive theorizing may “open the possibility of novel understandings,” as Charmaz emphasises (2014a, p. 243), but it does not guarantee that these understandings will have an epistemic status different from commonsensical explanations. By essentially relying on the researcher’s interpretive sensibility to develop theories, CGT’s epistemology is narrowing down the scope of knowledge to the best possible explanation one can come up with (see Miller and Fredericks 1999). The opposition between theory = preconception, on the one hand, and the absence (or lesser role) of theory = **no** preconception, on the other hand, is a false opposition from a rationalist position. The real opposition is between research based on an epistemological break to overcome common sense, and research discounting such a break and potentially confusing ready-made cognitive categories with concepts.

As Bourdieu et al. contend, “the illusory representation of the genesis of social facts according to which the social scientist can understand and explain these facts merely through ‘his own private reflection’ rests (…) on the presupposition of innate wisdom” (Bourdieu et al. 1991, p. 15), which is rooted in the false sense of familiarity with the social world. In this quote, Bourdieu et al. are raising a key question about the primary tool for knowledge production: innate wisdom or extant knowledge and theories? CGT advocates have not yet provided a satisfactory answer to this question. As we noted earlier, our understanding of Charmaz’s position is that while she favours induction, she acknowledges that researchers are not blank slates and, consequently, may (and likely do) build on extant knowledge. The relationship between these two ideas needs to be clarified.

It could be argued, against Bourdieu and colleagues, that professional researchers receive proper training and therefore hold the required expertise to conduct qualitative research, and that guidelines have been developed over the years to ensure the correct execution of qualitative research. Combined together, researchers’ expertise and guidelines constitute a solid basis for the creation of theories and knowledge that are not a mere reproduction of “innate wisdom.” While this argument may appear compelling at first glance, it raises more questions than answers. First, what is expertise? How can it be defined and whose definition should prevail over others (e.g. sociologists’ or psychologists’ definition?) The notion of expertise is too dependent on how it is defined, and by whom, to be used as a surrogate for the operationalisation of an epistemological break. Concerning guidelines, while they may be useful to novice qualitative researchers, they are not meant to address epistemological issues and therefore are of limited use for reflecting on epistemological questions. See Eakin and Mykhalovskiy (2003) for an insightful critique of guidelines in qualitative research.

### Subjectivity and relativism: tools for knowledge making

Another key feature of CGT is its support of relativism and subjectivity as tools for knowledge making, and its rejection of positivism and objectivity. We will quote Charmaz at length in order to present her position as accurately as possible. “Those of us who adhered to a relativist epistemology never concurred with grounding grounded theory in Glaser’s mid-twentieth-century positivism” (Charmaz 2008a, p. 401). “From a constructionist view objectivity is a questionable goal, and what researchers define as objective still reflects partial knowledge and particular perspectives, priorities, and positions. Subjectivities are embedded in data analysis, as well as in data collection” (Charmaz 2008a, p. 402). She adds, “Objectivists assume that data are self-evident and speak for themselves. Objectivists aim to generalize through abstractions that separate the completed grounded theory from the conditions and contingencies of its data collection and analysis. (…) Objectivists seek generalizations that provide explanations and predictions” (Charmaz 2008a, p. 402).

From a rationalist perspective, Charmaz’s position on objectivity is somewhat perplexing. Indeed, how is it possible to develop an understanding of the social world if the findings merely reflect the “perspective,” “position,” and “priorities” of the individual conducting the research? How can other scholars assess such understanding if the author of the study claims that his/her report reflects his/her personal view—or if the study is subjective by default? Some level of “abstraction” and “objectivity,” to use Charmaz’s words again, is needed in order to make claims about the world that can be discussed, refined, rejected, etc. Subjective accounts are useful for gaining access to one’s vision and experience of a given situation, but they are not meant to be an object of debate the way academic/scientific knowledge claims are. Taken altogether, Charmaz’s claims do not point toward moving away from the contingencies of the site or situation under examination and engaging in abstractions and theoretical generalizations.^2^

From a rationalist position, looking for regularities and patterns across time, social groups, local situations and micro events does not entail adhering to a positivistic epistemology and believing that knowledge is value-free, neutral and definitive. It means that one is seeking, through extant theoretical and methodological apparatuses, to understand fundamental social processes that may be at work in various settings, or unearthing new ones. To achieve such understanding, researchers need to display a dual gaze. They need to focus on the intricacies of the situation they are studying in order to disentangle and understand the micro and idiosyncratic social dynamics at work. At the same time, they need to take into account the underlying processes and mechanisms shaping micro/local events. As unique as these events may appear, they can be shaped by processes and mechanisms (such as organisational isomorphism, hidden curriculum, and struggle for cultural authority), also at work in other settings, thereby making them less distinctive than they seem to be at first glance. These two gazes do not stand in opposition to one another as Charmaz seems to suggest (2008a, p. 402), but are complementary. Theory building needs empirical grounding; understanding empirical situations requires theories. Arguing that abstraction (i.e., regularities and patterns) is an obstacle to the understanding of local situations is a claim rationalists would deem unproductive, as it leaves out key apparatuses (theory and extant knowledge) from the heuristic toolbox.

Another point of divergence between Charmaz and rationalists is that the latter do not equate objectivity with empiricism. For rationalists, a research report is not objective because the data presumably are, as Charmaz contends in her critique of objectivity (“Objectivists assume that data are self-evident and speak for themselves.”—Charmaz 2008a, p. 402). Rather, for rationalists, objectivity is a construct, inseparably social and scientific. It can be defined as an agreement on an explanation or a theory between researchers sharing a common set of concepts and methodological tools, with the aim of bringing to light hidden mechanisms and processes. This definition of objectivity implies that different ways of conducting scientific research will likely lead to the construction of different facts, i.e., different conceptual understandings of the phenomenon under study. It further entails the rejection of the belief that there can be a universal and permanent truth. As any agreement can be challenged by another emerging consensus, each is always necessarily provisional.^3^ According to the rationalists’ view on objectivity, Charmaz’s position needs to be nuanced as it is overly tilted toward empiricism.

## Concluding thoughts

While the usefulness of CGT for conducting qualitative research and understanding the perspective of social actors has been acknowledged by scholars in health professions education research and other research areas, the inductivist logic on which it draws—as advocated by Charmaz—raises questions concerning the nature of the knowledge yielded by this approach. As we have argued in this article, it is still unclear in what way CGT interpretative theory is not a duplication of everyday thinking expressed through meta-narratives. It is also unclear how the understanding of social phenomena can be refined if the use of inductive procedures logically implies the creation of a new theory each time a study is conducted.

From a practical standpoint, for practitioners and decision-makers in health professions education and other professional domains, the use of CGT-generated knowledge can be quite problematic. Indeed, how can decisions be made based on this knowledge if there are as many theories as there are studies, and if these theories are subjective and potentially contradictory? Given that research knowledge has become the mainstay of decision-making in contemporary societies, we are left wondering how CGT can play a significant role in this regard.

In this article, we have drawn on the rationalist epistemology originally developed by Bachelard (1985 [1934]) and taken up a few decades later by Bourdieu et al. (1991 [1968]) to look at the epistemological foundation of the CGT methods as defined by Kathy Charmaz. Other epistemologies, such as Bhaskar’s critical realism (1975) and Popper’s falsificationist methodology (2002 [1935]) would have shed a different light. The critical analysis of Bourdieu’s rationalism conducted by Vandenberghe (1999) is also enlightening as it insightfully deconstructs its premises and shows its connection with realism. It is our hope that scholars in the field of health professions education research will engage in this epistemological conversation and advance it in new directions.

## Footnotes

1 Various topics in health professions education research, such as competency-based medical education, professionalism and inter-professional education, could probably be seen as preconstructed objects. These topics have been primarily crafted by health profession organisations and academic administrators as educational issues to be addressed. The goal is not to gain a better understanding of social processes related to education but to correct, improve or implement new practices in health professions education. For this reason, these topics may be carrying hidden normative assumptions that impede research questions situated outside these assumptions. This is not the place to expand on how non-academically-based questions find their way into the scientific field, but further research would be helpful to understand the mechanisms allowing such processes to occur in health professions education research and how it affects the research enterprise.

2 While the general thrust of Charmaz’s epistemology points towards being critical of abstraction, some passages of her work puzzlingly suggest the opposite: “An abstract understanding of particular sites and situations can allow social constructionists to move from local worlds to a more general conceptual level. The close attention that social constructionist grounded theorists give their research problems builds the foundations for generic statements that they qualify according to particular temporal, social, and situational conditions” (Charmaz 2008a, p. 398). These statements seem to contradict her main argument promoting knowledge contextualisation. A potential explanation could be that she is defining “abstraction” here specifically as inductively derived theories in contrast with pre-existing and incrementally developed theories.

3 It is not uncommon to hear laboratory scientists themselves defining objectivity along similar terms. Many acknowledge that the laboratory environment is artificial and their results are interpretations based on hypotheses and extant knowledge (Albert et al. 2015). The two following quotes, from interviews with biomedical scientists, exemplify this. How you choose to ask a question, what you choose to report, how you choose to present it inherently changes the results in the way we view it. Data don’t speak by themselves because how we interpret them depends on the references upon which we interpret them (25BMS). Everything is a construction, there’s no such thing as truth. There’s only truth in religion. Everything’s is a construction consistent with a hypothesis, but you only have to do one experiment and show what you thought was wrong. So, truth is for believers, it’s not for scientists (10BMS) (Albert et al. 2015, p. 67).
